# Effects of Color and Light Intensity on the Foraging and Oviposition Behavior of *Culex pipiens* biotype *molestus* Mosquitoes

**DOI:** 10.3390/insects13110993

**Published:** 2022-10-29

**Authors:** Fanny Hellhammer, Hella Heidtmann, Fritjof Freise, Stefanie C. Becker

**Affiliations:** 1 Institute for Parasitology, Center for Infection Medicine, University of Veterinary Medicine Hannover, 30559 Hannover, Germany; 2 Research Center for Emerging Infections and Zoonoses, University of Veterinary Medicine Hannover, 30559 Hannover, Germany; 3 Department of Biometry, Epidemiology and Information Processing, University of Veterinary Medicine Hannover, 30559 Hannover, Germany

**Keywords:** *Culex pipiens* biotype *molestus*, colors, behavioral mechanism

## Abstract

**Simple Summary:**

Mosquitoes are involved in the transmission of many pathogens leading to diseases in humans and animals. Such so-called vector populations must be controlled to prevent and contain mosquito-borne disease outbreaks. For this reason, it is of great importance to understand the mechanisms by which mosquitoes locate hosts and choose oviposition sites. The present study investigated the effect of colors on foraging and oviposition behavior. Our study demonstrated that *Culex pipiens* biotype *molestus* mosquitoes are attracted by the color (for human eyes) red, if blue, green and yellow are provided as alternatives. We could also observe that the color black has a stronger attraction than red when mosquitoes are searching for food. This knowledge can be used as a new inexpensive and simple mosquito food preference-tracking method, as well as for improvement of oviposition traps for mosquitoes.

**Abstract:**

Mosquitoes are the most important vector of arboviruses; thus, controlling mosquito population is a key point for controlling these diseases. Two major factors that influence mosquito population size are the availability of blood hosts and suitable oviposition sites. Behavioral mechanisms by which *Culex pipiens* biotype *molestus* mosquitoes locate their hosts or oviposition sites are influenced by physical and chemical factors. The present study evaluated the impact of the colors (for human eyes) red, green, blue and yellow in combination with different light intensities on preferences for oviposition and foraging sites under laboratory conditions. We identified the color red as the overall favored color for both target behaviors, which was only surpassed by black as the foraging stimulus. Altogether, we described two new inexpensive and simple bioassays, which can be used as a mosquito-tracking method for behavioral tests and as an oviposition trap to monitor *Culex pipiens* biotype *molestus* populations.

## 1. Introduction

Mosquitoes (Diptera: Culicidae) are the most important vectors for viral and protozoan pathogens such as dengue or the causative agent of malaria *Plasmodium* spp.; thus, the control of vector populations is crucial to control outbreaks. Two major factors influencing mosquito population size are the availability of blood hosts and suitable oviposition sites [1]. Therefore, understanding the means by which mosquitoes locate foraging sites/hosts and choose oviposition sites can be crucial for efficient vector control. For example, the development of enhanced ovitraps for monitoring or the use of colors to control insect pests are interesting targets for novel malaria and arbovirus control strategies [2].

Most research on the host preferences of mosquitoes and location of oviposition sites focuses on olfactory perception by the mosquito. However, the visual perception of mosquitoes is needed to locate hosts, mates and resting sites but also food sources and oviposition sites [3]. It has been shown that the choice of a suitable oviposition site is not random. Rather, the attractiveness of oviposition sites depends on physical, chemical, biological and visual cues [4,5,6]. Numerous authors examined visual stimuli including shape, size, contrast, light intensity, texture and color as oviposition attractants for several different species including *Anopheles coluzzii* [2], *Aedes albopictus* and *Ae. polynesiensis* [7,8], *Ae. aegypti* [9], *Culex pipiens pallens* [10], *Cx. quinquefasciatus* [11], *Cx. annulirostris* and *Cx. pipiens* biotype *molestus* [12]. Furthermore, the significance of colored objects for foraging response or landing behavior was also repeatedly studied for *Ae. aegypti* [9,13,14,15], *Ae. albopictus*, *Anopheles quadrimaculatus* and *Cx. nigripalpus* [13]. The studies clearly demonstrated that different mosquito species of the same genus or different genera vary in their response to similar visual stimuli.

Mosquitoes of the *Culex pipiens* complex are of major interest due to their rapid expansion in the last years [16], being now native to Europe, America, Africa and Asia, [17]. These mosquitoes are known as vectors of many arboviruses including the West Nile virus (WNV) [18], Sindbis virus (SINV), Usutu virus (USUV) [16] or St. Louis encephalitis viruses (SLEV), as well as other pathogens such as avian malaria (*Plasmodium* spp.) and filarial worms [19] affecting humans and animals. Due to the emergence and outbreaks of diseases caused by these pathogens in the last decades [16], it is of great importance to understand, in addition to the ecology and physiology, the behavior of those transmitting mosquito species to enhance efficient control and population management. *Cx. pipiens* biotype *molestus* mosquitoes mate in restrained spaces and do not require a blood meal to produce the first egg raft [20]. They belong to the dichromatic insects, which perceive wavelengths from 300 nm (ultraviolet) to 650 nm (orange) and distinguish single colors and mixtures of colors [21,22]. It is already known that for gravid *Culex* spp., dyed water is more attractive for oviposition compared to undyed water [10,11], but only little is known about their behavior towards different-colored foraging sites. To investigate the effect of different colorations of oviposition and foraging sites on the behavior of *Culex* mosquitoes, several studies have developed different methods for laboratory and field conditions, such as dying distilled water [10] or ponds [23] with chemicals or using colored artificial flowers [24], experimental plants [25] or colored BGS traps [26]. In these studies, *Culex* spp. were attracted by dark colors such as black, blue or red. It could be shown that mosquito color vision systems such as wavelength sensitivity or the quantity of color receptor types are adapted to their activity peaks. For *Culex pipiens* spp., which show crepuscular and nocturnal foraging habits, it is known that they have a lower capacity for color distinction and sensitivity than diurnal species [27,28].

In this study, we aim to establish a bioassay for the analysis of visual stimuli and color preferences of *Cx. pipiens* biotype *molestus* regarding their oviposition and foraging behavior at different light intensities. For this purpose, we choose ink as a method of dyeing. Inks were one of the first methods and are still commonly used to mark insects’ bodies externally [19]. This external marking method is inexpensive but time-consuming. In contrast, oil-soluble dyes or other colors that can be ingested by insects and accumulate in their bodies [19] are an easy and fast way to mark them. Depending on the insect species, evaluation of the ingested color can be performed by a direct visual analysis or, if not externally visible, by crushing them on filter paper [19].

To this end, we set up a feeding assay using ink as food coloring to examine the impact of colors on their foraging by marking mosquitoes internally and oviposition site selection. With this knowledge, an improved vector control through better ovitraps as well as a simple, inexpensive laboratory method to track the foraging behavior of mosquitoes is possible.

## 2. Materials and Methods

### 2.1. Mosquito Colony

Laboratory strains of a *Culex pipiens* biotype *molestus* (Forskal, 1775) derived from mosquitoes collected in Northern Germany (Langenlehsten and Wendland) in 2012 and 2013 were maintained in rearing rooms at 25–26 °C, 45–65% RH and photoperiods of 16:8 (Light:Dark; separated by one hour of crepuscular periods; daylight 1600 lux (lx)). Larvae were reared in plastic basins (37 cm × 30 cm × 7 cm) filled with dechlorinated tap water from 3 to 5 cm depth and fed with TetraMin fish food (Tetra Werke, Melle, Germany) ad libitum. In order to fulfill this, fresh tap water was exposed to ambient air for at least 12 h to remove volatile components, such as chlorine. Pupae were separated from larvae and reared in plastic boxes until emergence of adult mosquitoes (12 cm × 9 cm × 7 cm). Adult *Culex pipiens* biotype *molestus* were maintained in Bugdorm cages (30 cm × 30 cm × 30 cm) and fed ad libitum with 8% (weight/volume) fructose solution supplemented with 0.5 g/L 4-aminobenzoic acid. Once a week, adults were blood-fed (cat or dog blood) after approximatively 24 h sugar water withdrawal.

### 2.2. Colors

Arctic-blue, strawberry-red, grass-green, neon-yellow and panther black-black ink (Seitz-Kreuznach, Bad Kreuznach, Germany) were used for oviposition and feeding assays. Depending on the bioassay and the color, between 2.8 mL and 21.5 mL of the ink was diluted in 1 L of dechlorinated tap water. For the oviposition bioassay, we also performed colored paperboard assay wrappings in the same colors as the ink-colored bioassay. For each color, the wavelength intensity was measured in the climate chamber lit with fluorescent light sources (Osram lumilux cool daylight l58 w/865) at a distance of 9 cm (from sensor to transparent 100 mL beakers with the colored solutions or paper wrapping) using a Gigahertz-Optik X4-DE-UN spectrophotometer calibrated with a grey card (4963 neutral Graukarte-Fotowand Technic, Sudwalde, Germany). The sensor was wrapped with a black paperboard, which formed a tunnel of 6.5 cm towards the 100 mL glass beaker. The paperboard ended 2.5 cm above the beaker (results in Figure S1). We also detected the absorbance of each ink color including green, red, yellow, blue and black using 50 μL of each ink–water solution and recording at 300 to 1000 nm via microplate reader (Tecan Spark, Tecan Group Ltd., Männedorf, Switzerland) (results in Figure S2).

### 2.3. Bioassays

Experiments were carried out in Bugdorm cages (30 cm × 30 cm × 30 cm) in windowless climate chambers without natural light. An average of around 200 mixed-sex mosquitoes, which were up to three-days-old and unfed, were used per experiment for all bioassays. Considering that counting while collecting mosquitoes is technically demanding and difficult at this scale, anesthesia (CO_2_ or cold) would be required to determine the number of mosquitoes before the start of an experiment. Therefore, the number of mosquitoes was determined after the end of each experiment so that the risk of side effects of anesthesia on mosquito sensory and behavior in the further experiments could be excluded. The number of mosquitoes used per experiment was estimated and counted only after the assays. The attractiveness of dyed oviposition and feeding sites was tested using ink-colored red, blue, green or yellow water. Black was only tested in a two-choice feeding assay. The arrangement of the four colored oviposition or feeding sites was changed in each replicate to avoid position-based effects. Initial tests were performed at 25–26 °C, 45–65% RH and 16:8 (Light:Dark including daylight at a light intensity of 1600 lx and a night period at almost 0 lx). In a further series, light conditions were changed: bioassays were repeated at constant light intensities of 130 lx or at 0 lx, respectively, to mimic twilight and night conditions. Every bioassay was repeated six times with independent mosquito batches.

#### 2.3.1. Oviposition Bioassay

To analyze the attractiveness of different colors for oviposition, 4.3 mL ink (green, red and yellow) diluted in 1 L dechlorinated tap water was used. The blue ink was used in a lower concentration (2.8 mL on 1 L dechlorinated tap water) to avoid a color too dark (for human eye) compared to the other offered colors.

The acceptance of ink solution as an oviposition option was investigated. For this purpose, the four tested colors (ink–water solution) and a beaker with nondyed tap water were offered at 0 lx. Three replicates with an average of 189 (range 173 to 207) mixed-sex mosquitoes were performed. After four days, the number of egg rafts and the number of females and males were counted.

In a second step, the four colored solutions were presented in transparent glass beakers (6.5 cm high and 4.5 cm diameter; 100 mL volume), excluding the possibility of lying eggs in nondyed tap water. To validate the results of colored water and to exclude an olfactory/chemical influence of the inks, the same transparent beakers were used, wrapped in colored paperboard (in the same colors (for the human eye) as in the ink-colored bioassay). On average, 303 (arithmetic mean; range 131 to 775) mixed-sex mosquitoes per replicate were used.

#### 2.3.2. Foraging Bioassay

Colored solutions for the feeding assays were prepared using 21.5 mL/L ink in 8% (*w*/*v*) fructose. The assays were conducted at 16:8 (L:D daylight at 1600 lx; constantly 130 lx; constantly 0 lx) and repeated six times using independent batches of mosquitoes. The mosquitoes had access to sugar-free uncolored water. The number of colored mosquitoes was recorded visually, differentiating the pure colors (red, blue, green, yellow and black), the mixed colors (fed from different colors) and the uncolored mosquitoes (not fed but with water access) after two days.

In a first step, we tested a four-choice assay. For this assay, an average of 279 (range from 114 to 522) mixed-sex mosquitoes per replicate were used. Of these, an average of 112 (range 38 to 194) were female.

In a second step, a two-choice assay, red-vs-blue, red-vs-green and red-vs-black, was used to confirm the results of the four-choice assay. For this assay, an average of 279 (range from 123 to 566) mixed-sex mosquitoes per replicate were used. Of these, an average of 123 (range 34 to 261) were female mosquitoes.

#### 2.3.3. Statistical Analyses

All statistical analyses were performed using R [29]. The influence of light conditions as well as paper- and ink-coloring of oviposition sites were analyzed using a multinomial logit model.

For the feeding assay, a multinomial logit model with lighting conditions as a factor modeled the number of mosquitoes with a certain color. All possible combinations of color and sex are expressed by the outcome categories. (e.g., “female and red”, “male and blue”). The models were fitted using the VGAM package [30]. Likelihood ratio tests, comparing the full model with a constraint one, were used to test the influence of the sex in the feeding assay, preceding pairwise comparisons. For testing the preference of a color compared to another (one-sided tests) and comparing choices of female and male mosquitoes (two-sided tests) in a post hoc analysis, Wald tests for linear combinations of the model parameters were used. These comparisons were conducted with the help of the multcomp package [31]. Bonferroni adjustment was used for tests on preference of a color and difference between sexes separately.

*p*-values smaller than 0.05 were assumed to indicate statistically significant results.

## 3. Results

### 3.1. Oviposition Bioassay

**Figure 1:**
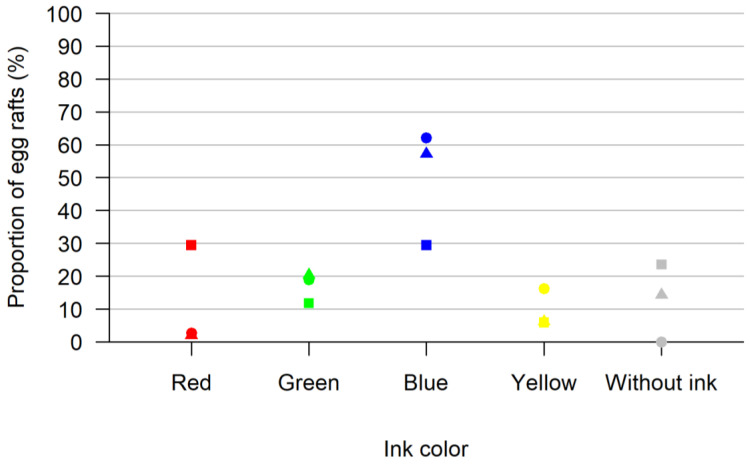
Results of the test for acceptance of ink solution as an oviposition option. Shown are the proportions of egg rafts laid in each colored oviposition site (red, green, blue, yellow and without ink) for all three replicates in percent. In each replicate, all colors were offered at once. Each replica is represented by a different form (square, triangle, circle).

We tested the acceptance of ink solution as an oviposition option. In total, 260 *Culex pipiens* biotype *molestus* females were tested (mixed with 308 male mosquitoes) laying 103 egg rafts. We observed a preference for the ink (92 of the 103 egg rafts laid) over the inkless water (11 of the 103 egg rafts laid). In total, 7 of the 103 eggs were laid in the red ink beaker, 19 were laid in the green ink beaker, 56 were laid in the blue ink beaker and 10 were laid in the yellow ink beaker (Figure 1).

In a second step, we tested the attractiveness of the colors excluding colorless oviposition sites. In the paperboard-wrapped oviposition site bioassay, 2067 females (out of 4649 tested mosquitoes), laying 640 egg rafts, were used. For the ink-colored water bioassay, 2812 *Culex pipiens* biotype *molestus* females (out of 6259 tested mosquitoes), laying 1402 egg rafts, were tested for their oviposition behavior. We observed an average number of egg rafts per female of 0.42 (Table 1). In both assay setups, the highest oviposition efficiency was noticed at constant twilight (130 lx), followed by the day–night cycle with max 1600 lx. The lowest oviposition efficiency was observed at 0 lx (0.25 and 0.26 egg rafts per female). Comparing both staining methods, on average females in the ink-colored water assay laid 0.19 egg rafts more than in the paperboard-wrapped beaker assay. This difference is even more pronounced at 130 lx setting with 0.73 egg rafts/female vs. 0.39 egg rafts/female in the ink-colored and paper-wrapped setup, respectively.

**Figure 2:**
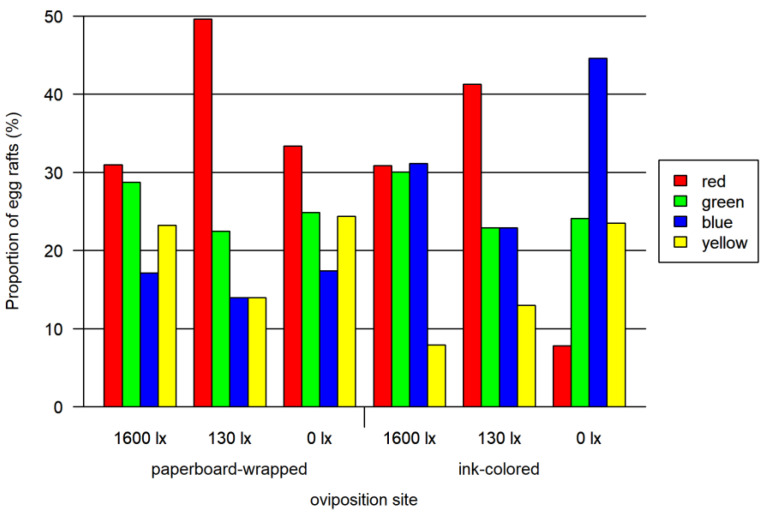
Results of paperboard-wrapped oviposition site bioassay and ink-colored oviposition site bioassay. Both tested at three light intensities. The 1600 lx light intensity was the maximal light intensity of a 16:8 h light cycle (Light:Dark; separated by one hour crepuscular periods; daylight 1600 lx), while the 130 lx and the 0 lx bioassay were at constant light intensity. The proportion of egg rafts (egg rafts per trial) is normalized for each combination of lighting and oviposition site.

The overall effect of light intensity, oviposition site (ink or paperboard) and their interaction was significant (likelihood ratio tests, all *p* < 0.0001). We detected a red preference in five of the six settings. Only the ink-colored oviposition site tested at 0 lx did not show the red preference. In detail, the results are as follows:

In the assay using paperboard-wrapped beakers as oviposition sites, red was preferred to all the other colors at twilight (130 lx, one-sided Wald test, adjusted *p* < 0.0001) and to blue at 0 lx (one-sided Wald test, adjusted *p* < 0.05), but no significant difference in other colors could be observed in full light (1600 lx). No further significant differences between the other colors were observed (Figure 2; overview in Tables S1 and S2).

In the assay using the ink-colored water in the oviposition site, the red preference was not as marked as for the paperboard-wrapped beakers. We detected significant preferences for colors using one-sided Wald tests: at 1600 lx red and green were significantly preferred to yellow (adjusted *p* < 0.0001). At 130 lx a significant red preference over all colors was identified (adjusted *p* < 0.0001) and a green and blue preference to yellow (adjusted *p* < 0.0012). At 0 lx blue (adjusted *p* < 0.0001), green (adjusted *p* < 0.01) and yellow (adjusted *p* < 0.011) were preferred to red. Blue was also preferred to yellow (adjusted *p* < 0.022) and green (adjusted *p* < 0.05). No further significant preferences were detected (Figure 2; overview in Tables S1 and S2).

### 3.2. Foraging Bioassay

**Figure 3:**
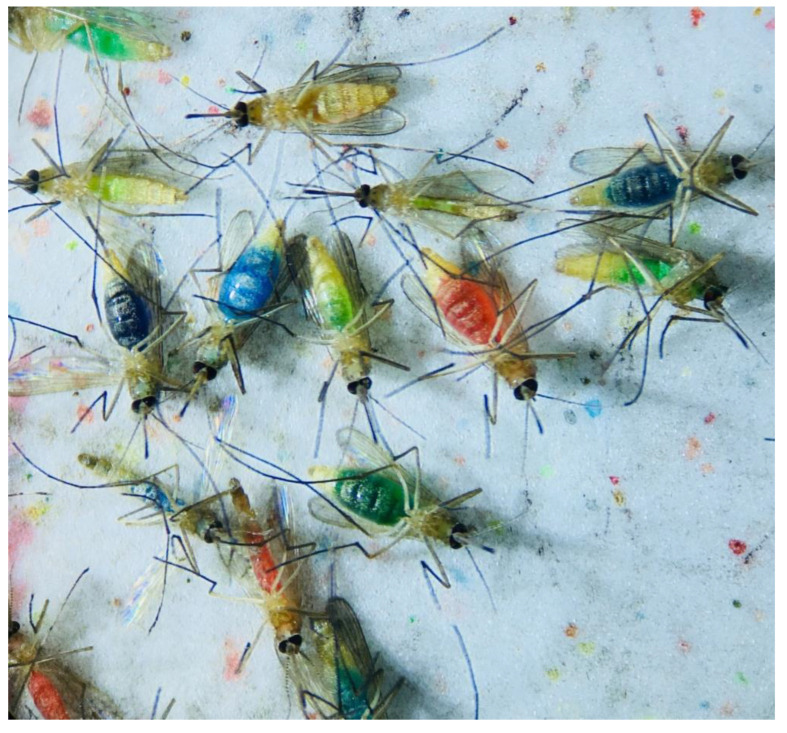
Excerpt from visual analyses of the mosquitoes of the four-choice feeding assay. The colors red, blue, green and yellow were offered simultaneously as ink–fructose–water solutions. Shown here is the wide range of color nuances of the original four offered colors. These results are due to ingesting different foraging amounts of the colored solution as well as the foraging from different colors.

Foraging assays were performed in two steps: the first step included a larger screening of colors for their preference, offering all colors (red, blue, green and yellow) in each replicate at once. For this part, we performed only a descriptive visual analysis of the data, excluding further statistical analyses due to the complexity of distinction of the generated color intensities and mixed colors of the abdomen of the mosquitoes, due to foraging in different amounts (lighter/darker color) and from different colors (Figure 3). This experiment allowed us to choose a refined color combination for the second step: offering only two suitable colors, resulting in a narrow and distinguishable range of color shades.

**Figure 4:**
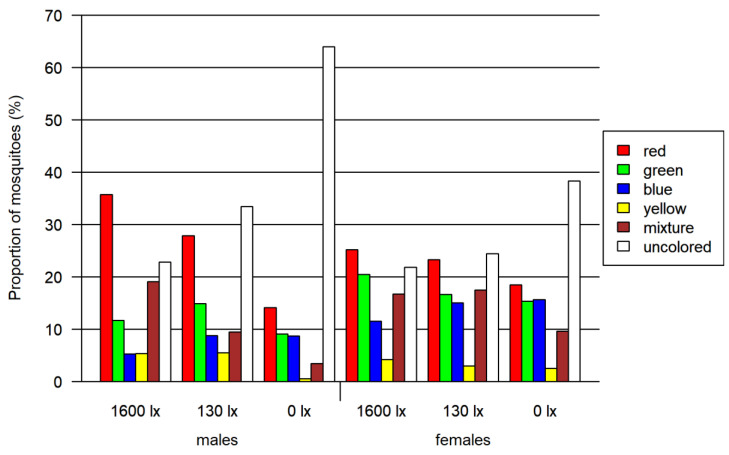
Results of the four-choice feeding bioassay. Left: results for females and males. Right: results only for females. Both tested at three light intensities. The 1600 lx light intensity was the maximum light intensity during a 16:8 h light cycle (Light:Dark; separated by one hour crepuscular periods; daylight at 1600 lx), while the 130 lx and the 0 lx bioassay were at constant light intensity. The proportion of mosquitoes is normalized by setting of lux.

In total, 2830 mosquitoes (including 1059 females) were tested in the four-choice foraging assay. A descriptive visual analysis was done for this data. We observed an overall preference for the colors red and green in every light condition, whereas blue and especially yellow color were avoided (Figure 3 and Table S3). In addition to the single-colored mosquito abdomen, between 6 and 19% of the mosquitoes had a mixed-colored abdomen due to feeding from more than one colored food source. A correlation between the light intensity and the color preference of mosquitoes is visible in Figure 4: with decreasing light intensity, the proportion of unfed/colorless and blue-colored mosquitoes increased while the proportion of mixed-, red- as well as green-colored mosquitoes decreased.

**Figure 5:**
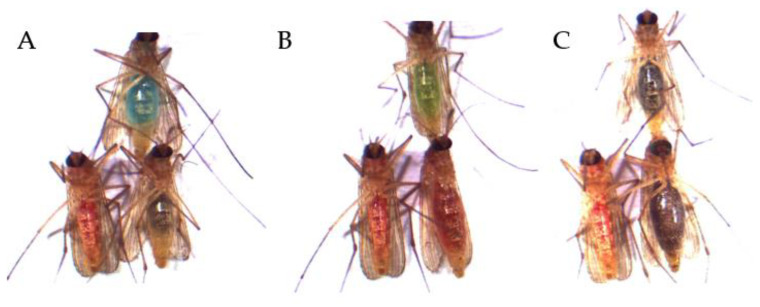
Excerpt from visual analyses of the mosquitoes of the two-choice feeding assays. The colors (**A**) red vs. blue, (**B**) red vs. green as well as (**C**) red vs. black were offered simultaneously as ink–fructose–water solutions. Shown here are the possible outcomes of each assay: lower left mosquito corresponds to the red abdominal staining after ingestion of the red solution; upper mosquito corresponds to the (**A**) blue staining, (**B**) green staining and (**C**) black abdominal staining after ingestion of the respective color; lower right mosquito corresponds with the mixture of the two other offered colors: (**A**) red–blue mixture, (**B**) red–green mixture and (**C**) red–black mixture.

**Figure 6:**
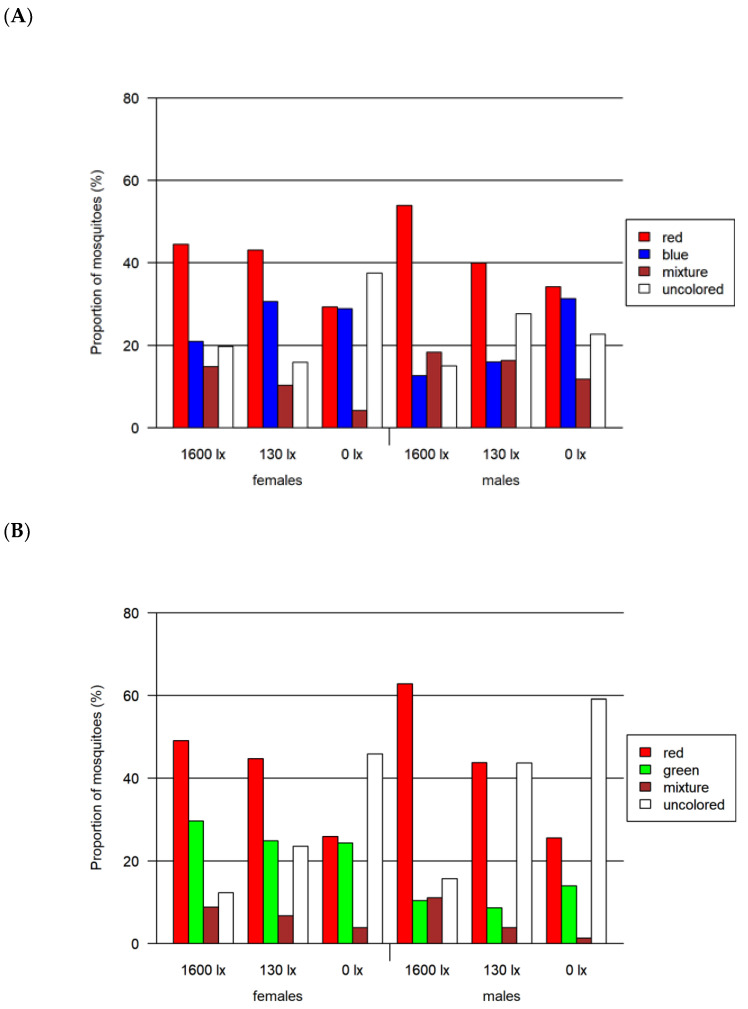

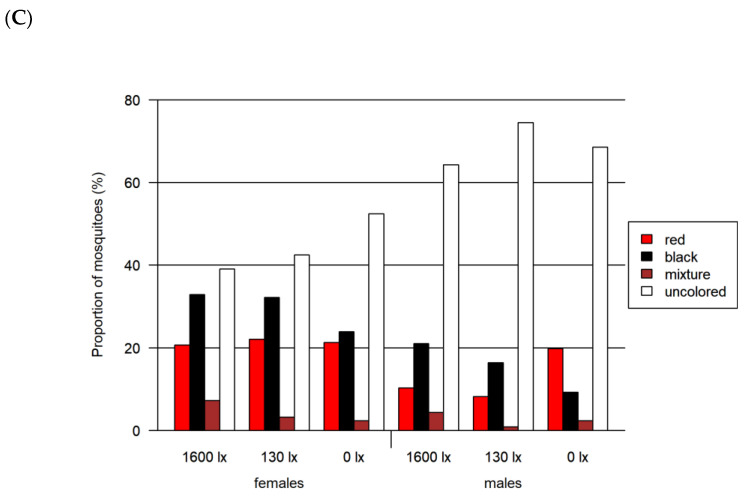
Results of the two-choice feeding bioassay. Left: results for females for (**A**) red-vs.-blue, (**B**) red-vs.-green and (**C**) red-vs.-black assays. Right: results only for males for the same assays. All assays were tested at three light intensities. The 1600 lx light intensity was the maximum light intensity of a 16:8 h light cycle (Light:Dark; separated by crepuscular periods; daylight at 1600 lx) while the 130 lx and the 0 lx bioassays were at constant light intensity. The given proportion of mosquitoes is normalized by setting of lux.

Given the apparent red preference, we tested this color against green, blue and black in subsequent two-choice assays to obtain a more detailed insight into color preferences. Furthermore, based on the four-choice assay results, we excluded yellow as a potential color preference and integrated black as a potential attractor. Altogether, in the two-choice red-vs.-blue assay, 6555 mosquitoes (including 2643 females) were investigated, 4554 mosquitoes (including 2173 females) in the two-choice red-vs.-green assay and 4606 mosquitoes (including 2102 females) in the two-choice red-vs.-black assay (Figure 5 and Figure 6).

In all three two-choice assays, male and female mosquitoes differed significantly in their choice of color (*p* < 0.0001 for all likelihood ratio tests). Overall, the proportion of uncolored mosquitoes was greater for lower light intensities (Table 2 and Figure 6), while the proportion of mixed-colored mosquitoes decreased with decreasing light intensity (Figure 6). The overall proportion of females that had ingested a colored solution (67.9% colored) was larger than for the males (57.4% colored). In all assays, the largest difference of around 20% between both sexes was detected at 130 lx.

(a) Two-choice assay red vs. blue (Figure 6A)

A significant preference for red over blue was found for both female and male mosquitoes at light intensities higher than 0 lx (adjusted *p* < 0.0001 in all cases). In this assay, we detected the highest proportion of 76.9% of colored mosquitoes as compared to both other two-choice assays. In contrast to the other two-choice assays, we identified the highest proportion of colored female mosquitoes at 130 lx. The proportion of mosquitoes with a certain color differed significantly when comparing males and females for most combinations of color and light intensity (adjusted *p* < 0.0001 in all cases, detailed results in Tables S4 and S5).

(b) Two-choice assay red vs. green (Figure 6B)

In the red-vs.-green two-choice assay, we found that red was preferred for all light intensities higher than 0 lx and additionally at 0 lx for male mosquitoes (adjusted *p* < 0.0001 in all cases). The proportion of colored mosquitoes was 66.1% in this assay. Significant differences between male and female mosquitoes were observed for green and uncolored (unfed) mosquitoes at all light intensities (adjusted *p* < 0.0008, except for uncolored at 1600 lx: adjusted *p* < 0.02) and for red at 1600 lx (adjusted *p* < 0.0001). The proportion of mixed-colored male and female mosquitoes did not differ significantly, but for 1600 lx and 0 lx the adjusted *p*-value was relatively small (adjusted *p* < 0.08).

(c) Two-choice assay red vs. black (Figure 6C)

In this red-vs.-black assay, the red preference was not observed (adjusted *p* < 0.0001). The proportions for red at 130 lx and 1600 lx were remarkably smaller than in the assays comparing red with blue and green. In this assay, we detected the lowest proportion of colored mosquitoes with 41.4%. In addition, the proportion of colored females (between 47.6 and 60.9%) was higher compared to males (25.6–35.8%).

## 4. Discussion

The attractiveness of colors to mosquitoes can be a useful tool in advanced vector control strategies [24,32]. In this study, we investigated several factors including light intensity, color of water in feeding and oviposition sites, as well as the color of the sites themselves as potential tools to manipulate the oviposition and feeding behavior of *Culex pipiens* biotype *molestus* mosquitoes. The light intensities were chosen to mimic full daylight (max. 1600 lx) in a 16:8 h Light:Dark cycle, persistent twilight (constant 130 lx) and, as a control, complete darkness (0 lx).

In a first step, we validated different colors in an oviposition assay using two different methods to apply the color to the oviposition site: either by coloring the waterbody or wrapping the transparent oviposition site with colored paper. Previous studies demonstrated that dark-colored oviposition sites are more attractive to *Culex* spp. For example, *Culex pipiens pallens* showed a significant blue preference, whereas [10] *Culex pipiens* biotype *molestus* showed a higher preference for red and *Culex annulirostris* for black-colored oviposition sites [12]. In a pond dye study, *Culex pipiens* revealed a preference for black color as compared to undyed water [23]. In our study using laboratory colonies of *Culex pipiens* biotype *molestus* mosquitoes derived from German mosquito populations, we obtained similar color preferences as described by Dhileepan [12], including the red preference and the avoidance of yellow. To exclude the olfactory stimuli emitting from the different inks from influencing our results, we also performed the experiment in complete darkness and a second set of experiments using colored paper-wrapped oviposition sites. In complete darkness (0 lx), the red preference was lost, indicating that the observed preference was based on visual rather than olfactory or other chemical stimuli. These results were also confirmed using the paperboard-wrapped beakers. In conclusion, we were able to demonstrate a red preference of *Culex pipiens* biotype *molestus* in oviposition site choice at daylight or twilight conditions. In contrast, the blue preference in the ink-colored group at 0 lx seems to be either an artefact or based on olfactory stimuli emitted from the blue ink, since we did not validate the result in the paperboard-wrapped beaker group. It has to be noted that at 0 lx conditions, a very low egg raft rate (<0.27 egg rafts per female) was observed in both assays. This indicates a low oviposition activity at complete darkness, which might make it more difficult to detect any preferences, olfactory or others. The highest egg raft numbers per female were detected in the ink-colored groups, which were on average 88% higher than the egg raft rates of the paperboard-wrapped groups in the same light conditions. The nature of this difference is not clear yet and might be subject to further studies. As a result of our study, the use of red ink-colored water in oviposition traps could be used to increase the egg deposition rate for *Culex pipiens* biotype *molestus* mosquitoes.

We also used the dyes from the oviposition experiment in a foraging assay. First, a four-choice assay with the colors blue, red, green and yellow was performed. Due to the fact that mosquitoes chose more than one color in 3–18% of the cases, many mixed colors with different intensities and nuances were generated, which were difficult to evaluate by eye. Therefore, this experiment was considered indicative, and the results were validated in two-choice assays. We selected two colors each in the two-choice assays that were easily distinguishable from each other and that generated an easily recognizable mixed color when mosquitoes fed from both colors. Since a red preference was evident in the visual analysis of the four-choice assay, we chose to use red and compare it with blue and green. In both assays, we confirmed the red preference at a light intensity above 0 lx for both sexes except for males in the red-vs-green-assay showing also the red preference at 0 lx. Previously published studies demonstrated that different mosquito species responded differently to similar visual stimuli and depending on their target (oviposition, resting, foraging) and their sex. For the mosquito *Aedes aegypti*, the color red was repetitively shown to be attractive for male and females. For example, Dieng et al. [24] showed a high female *Aedes aegypti* resting count for the color red followed by purple and blue, while males favored the color red followed by purple and yellow. Furthermore, Brett [33] reported a black and red preference and a yellow aversion for *Aedes aegypti* using colored clothes, and Kay et al. [14] obtained a similar red preference using cardboard traps. Besides *Aedes aegypti*, *Aedes tremulus* and *Culex quinquefasciatus* were sampled by this method. The compound eyes, called ommatidia, of *Culex* spp. are structurally similar to the ommatidia of *Aedes* spp. [34,35]. This could explain the similar color preferences (preference for red and black, aversion to white and yellow) in *Culex pipiens* and *Aedes albopictus* [26].

Since other than the apparent red preference, dark colors also were preferred by mosquitoes in other studies, we introduced the color black and compared it with red in a two-choice assay. Black was previously described as a potential attractant (landing, resting and oviposition studies) across different mosquito species [12,23,26]. We confirmed this black preference in our foraging assay over all light intensities and both sexes (except 0 lx). Interestingly, it is known that *Aedes aegypti* mosquitoes can perceive light in the range from about 323 nm to 621 nm [36], which means that they can perceive from violet through blue, green and yellow to the color orange. This indicates that they probably are not able to perceive the color red. In this study, however, the colors red and black differed in preference. This could suggest that the perception of the colors differs from human perception. Detected color preferences may be due to olfactory stimuli emanating from the ink. Since black has also been identified as an attractant in other studies, we assume that it is not primarily the olfactory stimulus that makes the color black attractive, but the color itself. Probably, the color red is perceived as less dark, but more grayish than the color black. Thus, it can be assumed that *Culex pipiens* biotype *molestus* mosquitoes prefer darker colors when foraging.

Furthermore, we were able to show that the light intensity during the experiments had a great influence on the results and that female and male mosquitoes reacted differently to the changes. Reducing the light intensity to 0 lx almost halved the number of fed mosquitoes compared to the 1600 lx group, which had a normal daily rhythm with light and dark phases. This result leads to the assumption that *Culex pipiens* biotype *molestus* also forage at night, but the activity decreases strongly in continuous darkness. In addition, a continuous twilight for 48 h led to a decreased foraging activity in almost all assays except the red-vs.-blue assay, where more females were colored than in the 1600 lx group. These results are contradictory to the results from field studies with *Culex pipiens* biotype *pipiens* [37,38], which described flight activity and host search primarily at night. However, if we compare the results with laboratory studies on ALAN (artificial light at night) with the same mosquito species as in this study [39], we can see many parallels: ALAN-exposed mosquitoes were less active during the extra-light phase which is equivalent to our 130 lx assays where less mosquitoes had taken the colored fructose meal; in this study, females were more active than males in nearly all phases regardless of treatment, which was explained by light-induced differences in sex-specific activity and which tendency (higher feeding rate) was also observed in our study. In addition to the observed sex-specific differences [39], the impact of the mating status might influence the foraging behavior. Since the mosquitoes used in our experiments were not separated by sex pre-eclosion, most of them will have mated before or during the experiment. Furthermore, females as well as males seem to need a complete light–dark rhythm to reach a peak of food-seeking activity. This could be explained by true resting phases, which do not seem to exist sufficiently at constant 130 lx, so that the feed intake performance decreases compared to the 1600 lx with light–dark phases. Furthermore, it has been shown that a disturbed circadian rhythm (such as the constant 130 lx assays in this study) leads to behavioral changes [40], which can explain the different results of the 1600 lx with the light–dark phases group compared to the constant 130 lx group. Due to the high proportion of colored mosquitoes in the 1600 lx (16:8 L:D) assay, we can support the statement that changing light intensity is an important trigger for activity.

In all experiments, we did not observe a toxic effect of the ingested ink. Larvae that hatched in the ink-colored beakers developed normally. Furthermore, the ingested ink was visible to the naked eye on the stained abdomens of the mosquitoes, so that an evaluation of the experiments required only little equipment. Ink also proved to be a suitable method of marking mosquitoes and tracking their behavior in this study. This method could be used to support two-choice assays. The evaluation of olfactory stimuli could be supported by the 0 lx assays where no color preference was detected (except red vs. blue).

To explore mosquito preferences for colors remains challenging due to targeted behavior (oviposition, foraging, landing, resting, etc.) and the variation across mosquito species and populations comprising their host preference, circadian rhythms and genetic and natural environment. In this study, we evaluated the impact of colors on the oviposition and foraging behavior of German *Culex pipiens* biotype *molestus*. We were able to validate previously reported color preferences of oviposition studies and extend knowledge to color preference in foraging and feeding assays. This study could lead not only to more effective trapping methods for *Culex pipiens* biotype *molestus*, but also to a new inexpensive and simple mosquito tracking method, which needs only little equipment to be evaluated.

## Figures and Tables

**Table 1:** Average number of egg rafts per female summarized for the six replications of each light intensity (1600 (16:8 L:D), constant 130 and constant 0 lx) and each assay (paperboard-wrapped beaker and ink-colored water assays).

Assay	Light Intensity in Lux	Egg Rafts Laid	Number of Tested Females	Average Number of Egg Rafts per Female	Mean
paperboard-wrapped beaker	1600	181	654	0.28	0.31
	130	258	669	0.39	
	0	201	744	0.27	
ink-colored water	1600	642	1374	0.47	0.50
	130	594	809	0.73	
	0	166	629	0.26	
**Mean**		340.3	813.2	0.42	

**Table 2:** Proportion of fed (colored) mosquitoes summarized for the six replicates of each light intensity, each assay (red vs. blue, red vs. green and red vs. black) and each sex.

Two-Choice Assay	Light Intensity (in Lux)	Number of Sucked (Colored) Mosquitoes (and Their Proportion)			Assay Mean
		Total	Female	Male	
**Red vs. blue**	1600 (16:8 L:D)	1325/1590 (83.4%)	373/455 (82.0%)	568/673 (84.4%)	4686/6092 (76.9%)
	130	1804/2321 (77.2%)	819/975 (84.0%)	788/1106 (71.2%)	
	0	1557/2181 (71.4%)	545/872 (62.5%)	1012/1309 (77.3%)	
**Red vs. green**	1600 (16:8 L:D)	1309/1526 (85.8%)	596/680 (87.6%)	713/846 (84.3%)	3216/4863 (66.1%)
	130	1171/1779 (65.8%)	644/843 (76.4%)	527/936 (56.3%)	
	0	736/1558 (47.2%)	405/748 (54.1%)	331/810 (40.9%)	
**Red vs. black**	1600 (16:8 L:D)	593/1274 (46.5%)	333/547 (60.9%)	260/727 (35.8%)	1908/4606 (41.4%)
	130	699/1782 (39.2%)	438/761 (57.6%)	261/1021 (25.6%)	
	0	616/1550 (39.7%)	378/794 (47.6%)	238/756 (31.5%)	
**Overall mean**		9229/14,859 (62.1%)	4531/6675 (67.9%)	4698/8184 (57.4%)	

## Data Availability

The datasets presented in this study are available on request from the corresponding author.

## Supplementary Materials

The following supporting information can be downloaded at: https://www.mdpi.com/article/10.3390/insects13110993/s1, Figure S1: Wavelength intensity of 100 mL of the colors red, blue, yellow and green in 100 mL-glass beaker compared to an empty beaker measured in a climate chamber lit with fluorescent light sources using a spectrometer. A shows the results for the paperboard-wrapped 100 mL beakers and B the results for the ink-colored 100 mL beakers. Figure S2: Absorbance of each ink solution (green, red, yellow, blue, black) recorded for a volume of 50 μL at 300 to 1000 nm via Tecan microplate reader. Table S1: One-sided statistical analysis of the results of paperboard-wrapped and ink-colored oviposition site bioassay. Both tested at three light intensities. The 1600 lx light intensity was the maximal light intensity of a 16:8 h light cycle (Light:Dark; separated by crepuscular periods; daylight 1600 lx), while the 130 lx and the 0 lx bioassay were at constant light intensity. The proportion of egg rafts is normalized for each combination of lighting and oviposition site. Table S2: Results of each replicate of the paperboard-wrapped oviposition site bioassay and ink-colored oviposition site bioassay. Both tested at three light intensities six times. The 1600 lx light intensity was the maximal light intensity of a 16:8 h light cycle (Light:Dark; separated by one-hour crepuscular periods; daylight 1600 lx), while the 130 lx and the 0 lx bioassay were at constant light intensity. Given are the number of egg rafts laid in each colored oviposition site (red, blue, yellow and green), as well as the total of female and male mosquitoes for each replicate. Table S3: Detailed results of each replicate of the four-choice feeding bioassay. Indicated are the light intensity at which the replicates took place, the total number of colored mosquitoes as well as the number of colored female mosquitoes for the respective colors (red, blue, green, yellow, mixture and uncolored) for each replicate. Furthermore, the number of used mosquitoes in total, separated also in the number of females and males for each replicate, are given. Table S4: One-sided statistical analysis using one-sided Wald tests of the results of the two-choice feeding bioassay: red vs. blue, red vs. green and red vs. black. All assays were tested at three light intensities. The 1600 lx light intensity was the maximal light intensity of a 16:8 h light cycle (Light:Dark; separated by crepuscular periods; daylight 1600 lx), while the 130 lx and the 0 lx bioassay were at constant light intensity. The given proportion of mosquitoes is normalized by the setting of Lux. Results were given for females and males separately. Table S5: Two-sided statistical analysis of the results of the two-choice feeding bioassay: red vs. blue, red vs. green and red vs. black. All assays were tested at three light intensities. The 1600 lx light intensity was the maximal light intensity of a 16:8 h light cycle (Light:Dark; separated by crepuscular periods; daylight 1600 lx), while the 130 lx and the 0 lx bioassay were at constant light intensity. The given proportion of mosquitoes is normalized by the setting of Lux. Results were given for each color of each color combination including the mixture of both colors and uncolored mosquitoes separately.
