# Supplementary material for: Effects of Color and Light Intensity on the Foraging and Oviposition Behavior of Culex pipiens biotype molestus Mosquitoes
Source: Insects. 2022 Oct 29;13(11):993. doi: 10.3390/insects13110993 (PMC9698050; doi:10.3390/insects13110993)
Supplement: Supplementary file 1 [file insects-13-00993-s001.zip › Supplementary Figure S1 wavelength.pdf]

A

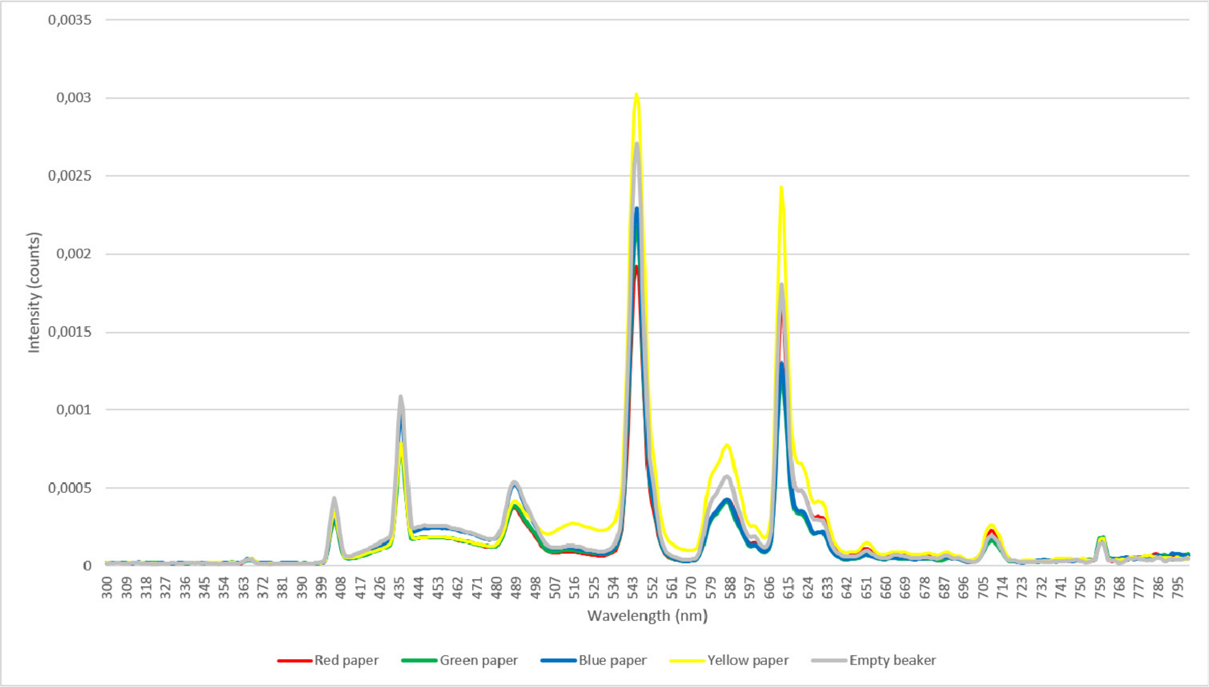

B

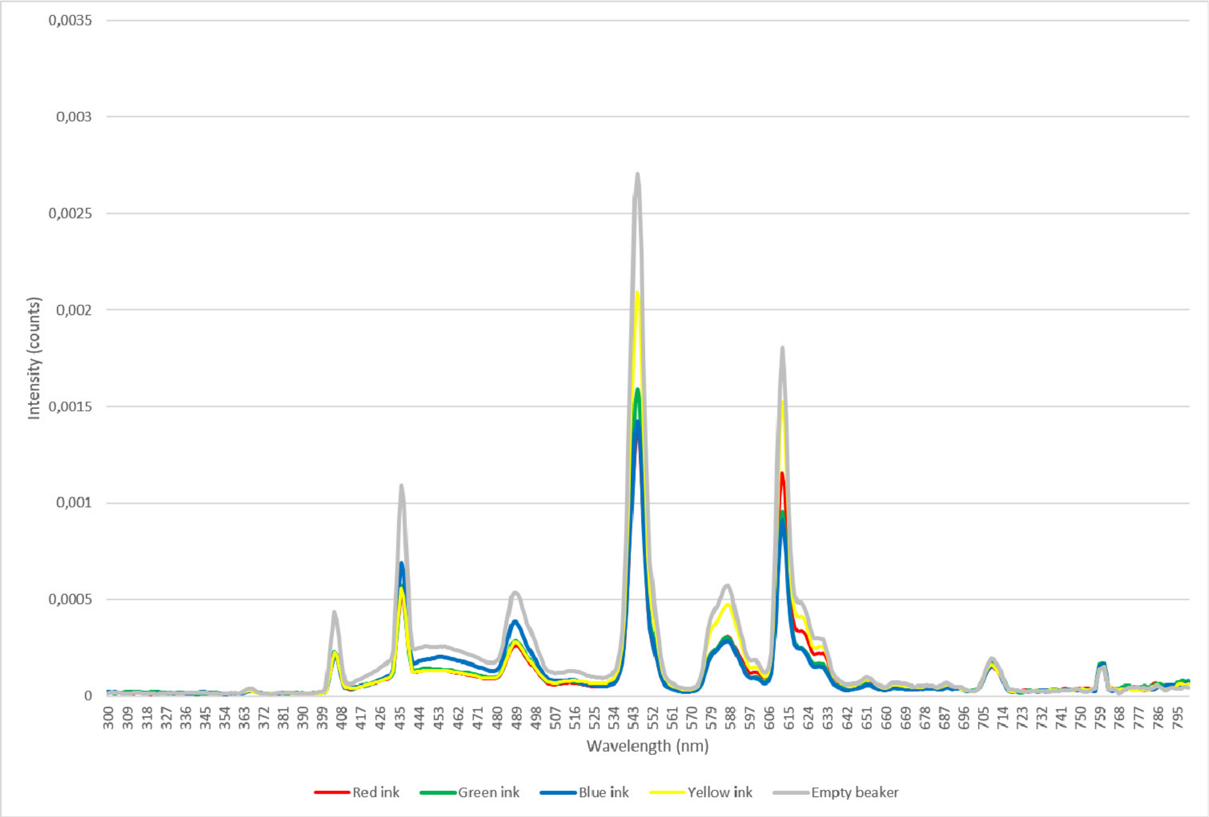

Figure S1 Wavelength intensity of 100 ml of the colors red, green, blue and yellow in 100 ml-glass beaker compared to an empty beaker measured in a climate chamber lit with fluorescent light sources using a spectrometer. A shows the results for the paperboard-wrapped 100 ml-beakers and B the results for the ink-colored 100 ml-beakers.
