# Supplementary material for: Effects of Color and Light Intensity on the Foraging and Oviposition Behavior of Culex pipiens biotype molestus Mosquitoes
Source: Insects. 2022 Oct 29;13(11):993. doi: 10.3390/insects13110993 (PMC9698050; doi:10.3390/insects13110993)
Supplement: Supplementary file 1 [file insects-13-00993-s001.zip › Supplementary Figure S2 Absorbance.pdf]

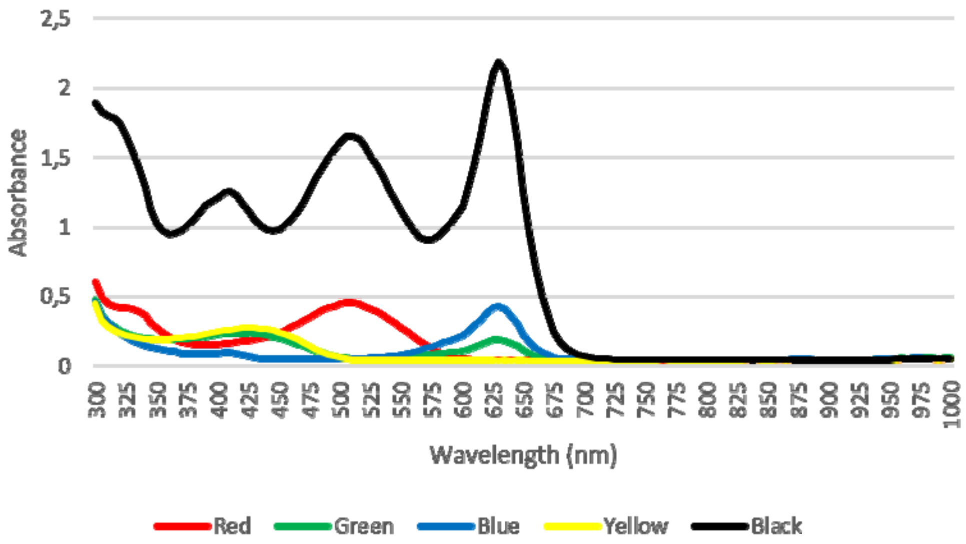

Figure S2 Absorbance of each ink-solution (red, green, blue, yellow and black) recorded for a volume of 50 µl at 300 to 1000 nm via Tecan microplate reader.
