# Supplementary material for: Effects of Color and Light Intensity on the Foraging and Oviposition Behavior of Culex pipiens biotype molestus Mosquitoes
Source: Insects. 2022 Oct 29;13(11):993. doi: 10.3390/insects13110993 (PMC9698050; doi:10.3390/insects13110993)
Supplement: Supplementary file 1 [file insects-13-00993-s001.zip › Supplementary Table S1_Results of one-sided Wald tests .pdf]

**Table S1:** Results of one-sided Wald tests testing for preference of a color in the results of paperboard-wrapped and ink-colored oviposition site bioassay. Both tested at three light intensities. The 1600 lx light intensity was the maximal light intensity of an 16:8 hours light cycle (Light:Dark; separated by crepuscular periods; daylight 1600 lx) while the 130 lx and the 0 lx bioassay were at constant light intensity. The proportion of egg rafts is normalized for each combination of lighting and oviposition site. Results are given for females and males separately. Adjusted p-values are calculated using Bonferroni adjustment.

| Dying method | Light intensity in Lux | Null hypothesis | Estimate | Std. error | t-statistic | p-value | adjusted P-value | significance |
|---|---|---|---|---|---|---|---|---|
| ink-colored | 0 | Blue <= Green | -0,61518564 | 0,19624861 | -3,13472613 | 0,00086007 | 0,03096263 | * |
| ink-colored | 0 | Blue <= Yellow | -0,64050345 | 0,19787536 | -3,23690353 | 0,00060417 | 0,02175017 | * |
| ink-colored | 0 | Red >= Blue | -1,73911574 | 0,30072328 | -5,78310983 | 3,6666E-09 | 1,32E-07 | *** |
| ink-colored | 0 | Red >= Green | -1,1239301 | 0,31925041 | -3,52052832 | 0,00021534 | 0,00775238 | ** |
| ink-colored | 0 | Red >= Yellow | -1,09861229 | 0,32025297 | -3,43045154 | 0,00030129 | 0,0108464 | * |
| ink-colored | 0 | Yellow >= Green | -0,02531781 | 0,22503553 | -0,11250582 | 0,45521118 | 1 | n.s. |
| ink-colored | 130 | Blue <= Yellow | -0,56884946 | 0,14261766 | -3,98863264 | 3,3228E-05 | 0,00119619 | ** |
| ink-colored | 130 | Blue >= Green | -1,4569E-12 | 0,12126794 | -1,2014E-11 | 0,5 | 1 | n.s. |
| ink-colored | 130 | Red <= Blue | -0,58860332 | 0,10693266 | -5,50442975 | 1,8518E-08 | 6,6666E-07 | *** |
| ink-colored | 130 | Red <= Green | -0,58860332 | 0,10693269 | -5,50442855 | 1,8518E-08 | 6,6666E-07 | *** |
| ink-colored | 130 | Red <= Yellow | -1,15745279 | 0,1306464 | -8,85943089 | 4,0212E-19 | 1,4476E-17 | *** |
| ink-colored | 130 | Yellow >= Green | -0,56884946 | 0,14261768 | -3,98863215 | 3,3228E-05 | 0,0011962 | ** |
| ink-colored | 1600 | Blue >= Green | 0,03562718 | 0,10090266 | 0,35308462 | 0,6379875 | 1 | n.s. |
| ink-colored | 1600 | Blue >= Yellow | 1,36649173 | 0,15686887 | 8,71104462 | 1 | 1 | n.s. |
| ink-colored | 1600 | Red <= Blue | 0,01005034 | 0,10025221 | 0,10025052 | 0,53992728 | 1 | n.s. |
| ink-colored | 1600 | Red <= Green | -0,02557684 | 0,10115262 | -0,25285398 | 0,40019053 | 1 | n.s. |
| ink-colored | 1600 | Red <= Yellow | -1,3564414 | 0,15702977 | -8,63811635 | 2,8574E-18 | 1,0287E-16 | *** |
| ink-colored | 1600 | Yellow >= Green | -1,33086456 | 0,15744583 | -8,45284086 | 1,4215E-17 | 5,1174E-16 | *** |
| paperboard-wrapped | 0 | Blue <= Yellow | 0,33647224 | 0,22131336 | 1,52034311 | 0,93578762 | 1 | n.s. |
| paperboard-wrapped | 0 | Blue >= Green | -0,35667494 | 0,22038925 | -1,61838627 | 0,05278969 | 1 | n.s. |
| paperboard-wrapped | 0 | Red <= Blue | -0,64934456 | 0,20855888 | -3,11348314 | 0,00092447 | 0,03328075 | * |
| paperboard-wrapped | 0 | Red <= Green | -0,29266961 | 0,18688329 | -1,56605553 | 0,05866781 | 1 | n.s. |

| | | | | | | | | |
|---|---|---|---|---|---|---|---|---|
| paperboard-wrapped | 0 | Red <= Yellow | -0,31287232 | 0,18797219 | -1,66446072 | 0,0480102 | 1 | n.s. |
| paperboard-wrapped | 0 | Yellow >= Green | -0,02020271 | 0,20101781 | -0,10050208 | 0,45997287 | 1 | n.s. |
| paperboard-wrapped | 130 | Blue >= Green | -0,47692407 | 0,21217718 | -2,24776329 | 0,01229564 | 0,44264319 | n.s. |
| paperboard-wrapped | 130 | Blue >= Yellow | -5,4817E-16 | 0,23570226 | -2,3257E-15 | 0,5 | 1 | n.s. |
| paperboard-wrapped | 130 | Red <= Blue | -1,26851133 | 0,18865386 | -6,72401471 | 8,8392E-12 | 3,1821E-10 | *** |
| paperboard-wrapped | 130 | Red <= Green | -0,79158725 | 0,15828417 | -5,00105141 | 2,8509E-07 | 1,0263E-05 | *** |
| paperboard-wrapped | 130 | Red <= Yellow | -1,26851133 | 0,18865386 | -6,72401473 | 8,8392E-12 | 3,1821E-10 | *** |
| paperboard-wrapped | 130 | Yellow >= Green | -0,47692407 | 0,21217718 | -2,24776329 | 0,01229564 | 0,44264318 | n.s. |
| paperboard-wrapped | 1600 | Blue >= Green | -0,51725651 | 0,22691143 | -2,27955244 | 0,01131712 | 0,40741643 | n.s. |
| paperboard-wrapped | 1600 | Blue >= Yellow | -0,30368241 | 0,23678606 | -1,28251811 | 0,09983048 | 1 | n.s. |
| paperboard-wrapped | 1600 | Red <= Blue | -0,59136449 | 0,22386346 | -2,64163018 | 0,0041254 | 0,14851456 | n.s. |
| paperboard-wrapped | 1600 | Red <= Green | -0,07410797 | 0,19258208 | -0,38481239 | 0,35018821 | 1 | n.s. |
| paperboard-wrapped | 1600 | Red <= Yellow | -0,28768207 | 0,20412423 | -1,40934802 | 0,07936614 | 1 | n.s. |
| paperboard-wrapped | 1600 | Yellow >= Green | -0,2135741 | 0,2074624 | -1,02945932 | 0,15163194 | 1 | n.s. |
