# Supplementary material for: Effects of Color and Light Intensity on the Foraging and Oviposition Behavior of Culex pipiens biotype molestus Mosquitoes
Source: Insects. 2022 Oct 29;13(11):993. doi: 10.3390/insects13110993 (PMC9698050; doi:10.3390/insects13110993)
Supplement: Supplementary file 1 [file insects-13-00993-s001.zip › Supplementary Table S2 Results of oviposition bioassay .pdf]

Table S2: Results of each replicate of the paperboard-wrapped oviposition site bioassay and ink-colored oviposition site bioassay. Both tested at three light intensities six times. The 1600-lx-light intensity was the maximal light intensity of a 16:8 hours light cycle (Light:Dark; separated by one-hour crepuscular periods; daylight 1600 lx) while the 130 lx and the 0 lx bioassay were at constant light intensity. Given are the number of egg rafts laid in each colored oviposition site (red, blue, yellow, and green), as well as the total of female and male mosquitoes for each replicate.

Oviposition site	Lux (lx)	Egg rafts laid in red	Egg rafts laid in blue	Egg rafts laid in yellow	Egg rafts laid in green	Total of female mosquitoes	Total of male mosquitoes	Total of mosquitoes
paperboard-wrapped	1600	0	9	12	12	90	160	250
	1600	6	1	2	1	83	105	188
	1600	10	9	7	11	76	102	178
	1600	8	1	2	1	196	244	440
	1600	9	7	11	10	100	114	214
	1600	23	4	8	17	109	179	288
paperboard-wrapped	130	47	9	8	17	117	87	204
	130	10	5	7	7	82	137	219
	130	17	3	3	18	136	188	324
	130	50	14	14	11	202	157	359
	130	2	5	3	1	70	106	176
	130	2	0	1	4	62	76	138
paperboard-wrapped	0	11	6	7	1	103	83	186
	0	12	15	17	17	106	219	325
	0	27	5	10	7	152	162	314
	0	10	4	4	16	138	189	327
	0	3	4	8	4	141	160	301
	0	4	1	3	5	104	114	218
ink-colored	1600	13	27	6	29	182	219	401
	1600	25	24	6	30	300	179	479
	1600	24	13	1	10	112	171	283
	1600	51	64	11	35	305	256	561
	1600	7	10	2	13	42	257	299
	1600	78	62	25	76	433	342	775
ink-colored	130	66	51	30	35	184	236	420
	130	9	21	3	22	105	126	231
	130	37	29	15	29	113	207	320
	130	56	16	6	15	117	321	438
	130	65	13	4	20	207	68	275
	130	12	6	19	15	83	258	341
ink-colored	0	8	21	4	10	150	171	321
	0	3	16	8	14	74	146	220
	0	0	3	0	2	62	69	131
	0	2	21	21	7	188	178	366
	0	0	2	5	1	48	85	133
	0	0	11	1	6	107	158	265
