# Supplementary material for: Effects of Color and Light Intensity on the Foraging and Oviposition Behavior of Culex pipiens biotype molestus Mosquitoes
Source: Insects. 2022 Oct 29;13(11):993. doi: 10.3390/insects13110993 (PMC9698050; doi:10.3390/insects13110993)
Supplement: Supplementary file 1 [file insects-13-00993-s001.zip › Supplementary Table S3 Detailed results of each replicate of the four-choice feeding bioassay.pdf]

Table S3: Detailed results of each replicate of the four-choice feeding bioassay. Indicated are the light intensity at which the replicates took place, the total number of colored mosquitoes as well as the number of colored female mosquitoes for the respective colors (red, blue, green, yellow, mixture and uncolored) for each replicate. Furthermore, the number of used mosquitoes in total, separated also in the number of females and males for each replicate are given.

Lux (lx)	Red total	Red females	Blue total	Blue females	Green total	Green females	Yellow total	Yellow females	Mixture total	Mixture females	Uncolored total	Uncolored females	Mosquitoes total	Mosquito females	Mosquito males
1600	57	16	5	3	11	7	0	0	6	3	35	9	114	38	76
1600	41	18	8	6	18	13	2	2	6	6	65	15	140	60	80
1600	47	15	25	11	9	7	1	1	13	6	71	22	166	62	104
1600	87	8	33	14	68	16	31	8	78	11	82	22	379	79	300
1600	98	34	7	5	52	30	23	4	55	23	32	23	267	119	148
1600	101	22	21	13	38	19	10	4	87	26	16	7	273	91	182
130	64	35	31	25	8	8	0	0	26	22	75	21	204	111	93
130	65	20	16	13	9	7	0	0	42	37	118	33	250	110	140
130	89	37	29	23	15	12	0	0	38	27	148	45	319	144	175
130	102	35	47	20	151	51	59	13	52	23	111	52	522	194	328
130	74	26	23	17	35	22	5	3	21	10	21	14	179	92	87
130	50	9	48	7	49	16	13	5	39	3	35	5	234	45	189
0	15	9	37	17	19	14	0	0	8	8	144	39	223	87	136
0	24	13	31	13	25	15	0	0	9	7	137	52	226	100	126
0	24	17	22	17	19	12	0	0	5	4	150	76	220	126	94
0	115	42	22	11	50	19	1	0	20	8	127	64	335	144	191
0	27	8	32	18	32	17	11	8	16	6	48	5	166	62	104
0	47	40	41	33	41	30	11	10	39	34	216	31	395	178	217
