# Supplementary material for: Effects of Color and Light Intensity on the Foraging and Oviposition Behavior of Culex pipiens biotype molestus Mosquitoes
Source: Insects. 2022 Oct 29;13(11):993. doi: 10.3390/insects13110993 (PMC9698050; doi:10.3390/insects13110993)
Supplement: Supplementary file 1 [file insects-13-00993-s001.zip › Supplementary Table S4 One-sided statistical analysis of the results of the two-choice feeding bioassay_2022-09-20_FF.pdf]

Table S4: Results of one-sided Wald tests testing for preference of red in the two-choice feeding bioassays: red vs. blue, red vs. green and red vs. black. All assays were tested at three light intensities. The 1600 lx light intensity was the maximal light intensity of an 16:8 hours light cycle (Light:Dark; separated by crepuscular periods; daylight 1600 lx) while the 130 lx and the 0 lx bioassay were at constant light intensity. Results are given for females and males separately. Adjusted p-values are calculated using Bonferroni adjustment.

Two-choice assay	Light intensity in Lux	Sex	Null hypothesis	Estimate	Std. error	t-statistic	p-value	Adjusted p-value	significance
red vs blue	0	female	Red <= Blue	-0,01574836	0,08873841	-0,17746945	0,42956983	1	n.s.
red vs blue	130	female	Red <= Blue	-0,34126632	0,072335	-4,7178589	1,1917E-06	7,1502E-06	***
red vs blue	1600	female	Red <= Blue	-0,75423487	0,10759985	-7,0096275	1,1948E-12	7,1686E-12	***
red vs blue	0	male	Red <= Blue	-0,08863607	0,06834598	-1,29687329	0,09733739	0,58402437	n.s.
red vs blue	130	male	Red <= Blue	-0,91330119	0,08349357	-10,938581	3,7685E-28	2,2611E-27	***
red vs blue	1600	male	Red <= Blue	-1,44456327	0,0994324	-14,5280946	4,0213E-48	2,4128E-47	***
red vs green	0	female	Red <= Green	-0,06385147	0,10319469	-0,61874763	0,26804131	1	n.s.
red vs green	130	female	Red <= Green	-0,58513766	0,08610706	-6,79546655	5,3981E-12	3,2389E-11	***
red vs green	1600	female	Red <= Green	-0,5028733	0,08913196	-5,64189627	8,4094E-09	5,0456E-08	***
red vs green	0	male	Red <= Green	-0,60533097	0,11696356	-5,17538071	1,1372E-07	6,8234E-07	***
red vs green	130	male	Red <= Green	-1,62170801	0,12159238	-13,3372506	7,0274E-41	4,2164E-40	***
red vs green	1600	male	Red <= Green	-1,79742521	0,11509507	-15,6168738	2,794E-55	1,6764E-54	***
red vs black	0	female	Red <= Black	0,11712536	0,10573703	1,1077043	0,86600523	1	n.s.
red vs black	130	female	Red <= Black	0,37729423	0,10016992	3,76654207	0,99991724	1	n.s.
red vs black	1600	female	Red <= Black	0,46556903	0,1200213	3,87905326	0,99994757	1	n.s.
red vs black	0	male	Red <= Black	-0,76214005	0,14474938	-5,26523885	7,0004E-08	4,2002E-07	***
red vs black	130	male	Red <= Black	0,69314718	0,13363062	5,18703854	0,99999989	1	n.s.
red vs black	1600	male	Red <= Black	0,71294981	0,14095844	5,05787245	0,99999979	1	n.s.
