# Supplementary material for: Effects of Color and Light Intensity on the Foraging and Oviposition Behavior of Culex pipiens biotype molestus Mosquitoes
Source: Insects. 2022 Oct 29;13(11):993. doi: 10.3390/insects13110993 (PMC9698050; doi:10.3390/insects13110993)
Supplement: Supplementary file 1 [file insects-13-00993-s001.zip › Supplementary Table S5 Two-sided statistical analysis of the results of the two-choice feeding bioassay_2022-09-20_FF.pdf]

Table S5: Results of two-sided Wald tests for comparing choices of female and male mosquitoes in the two-choice feeding bioassays: red vs. blue, red vs. green and red vs. black. All assays were tested at three light intensities. The 1600 lx light intensity was the maximal light intensity of an 16:8 hours light cycle (Light:Dark; separated by crepuscular periods; daylight 1600 lx) while the 130 lx and the 0 lx bioassay were at constant light intensity. Results are given for each color of each color combination including the mixture of both colors and uncolored mosquitoes separately. Adjusted p-values are calculated using Bonferroni adjustment.

Two-choice assay	Light intensity in Lux	Color	Null hypothesis	Estimate	Std. error	t-statisitic	p-value	adjusted P-value	significance
red vs blue	0	red	female = male	0,55961579	0,0783479	7,14270273	9,1505E-13	1,0981E-11	***
red vs blue	0	blue	female = male	0,48672807	0,08004551	6,08064153	1,197E-09	1,4364E-08	***
red vs blue	0	mixture	female = male	1,42603469	0,18308606	7,7888762	6,6613E-15	7,9936E-14	***
red vs blue	0	uncolored	female = male	0,09622803	0,08015669	1,20049909	0,22994556	1	n.s.
red vs blue	130	red	female = male	0,08537961	0,06457598	1,32215736	0,18611576	1	n.s.
red vs blue	130	blue	female = male	-0,48665526	0,08962963	-5,42962466	5,6473E-08	6,7767E-07	***
red vs blue	130	mixture	female = male	0,62252963	0,1181861	5,26736775	1,3839E-07	1,6607E-06	***
red vs blue	130	uncolored	female = male	0,71352634	0,09361675	7,62178116	2,5091E-14	3,0109E-13	***
red vs blue	1600	red	female = male	0,67445505	0,07476965	9,02043914	0	0	***
red vs blue	1600	blue	female = male	-0,01587335	0,12599218	-0,12598678	0,89974239	1	n.s.
red vs blue	1600	mixture	female = male	0,69314718	0,12909951	5,36909227	7,9134E-08	9,4961E-07	***
red vs blue	1600	uncolored	female = male	0,20972053	0,12284154	1,70724442	0,08777663	1	n.s.
red vs green	0	red	female = male	0,06486063	0,09992776	0,64907524	0,51628975	1	n.s.
red vs green	0	green	female = male	-0,47661887	0,1197667	-3,97956075	6,9043E-05	0,00082851	***
red vs green	0	mixture	female = male	-0,96940056	0,35410711	-2,73759132	0,00618909	0,07426912	n.s.
red vs green	0	uncolored	female = male	-0,33397015	0,07073284	-4,72157138	2,3403E-06	2,8084E-05	***
red vs green	130	red	female = male	0,08391197	0,07135506	1,17597781	0,23960375	1	n.s.
red vs green	130	green	female = male	-0,95265838	0,13079597	-7,28354506	3,2507E-13	3,9009E-12	***
red vs green	130	mixture	female = male	-0,45953233	0,2128882	-2,15856174	0,03088418	0,37061021	n.s.
red vs green	130	uncolored	female = male	0,72041033	0,08642983	8,33520516	0	0	***
red vs green	1600	red	female = male	0,46362103	0,06983732	6,6385857	3,1671E-11	3,8005E-10	***
red vs green	1600	green	female = male	-0,83093088	0,12772678	-6,50553357	7,7418E-11	9,2902E-10	***

red vs green	1600	mixture	female = male	0,44895022	0,16524214	2,71692331	0,00658919	0,07907025	n.s.
red vs green	1600	uncolored	female = male	0,45953233	0,13936843	3,29724841	0,00097637	0,01171645	*
red vs black	0	red	female = male	-0,11926342	0,11217766	-1,06316552	0,28770689	1	n.s.
red vs black	0	black	female = male	-0,99852883	0,13981729	-7,14166933	9,2215E-13	1,1066E-11	***
red vs black	0	mixture	female = male	-0,05406722	0,32891757	-0,16437925	0,8694326	1	n.s.
red vs black	0	uncolored	female = male	-0,21928998	0,06583577	-3,33086386	0,00086577	0,01038923	*
red vs black	130	red	female = male	-0,69314718	0,13363062	-5,18703854	2,1366E-07	2,564E-06	***
red vs black	130	black	female = male	-0,37729423	0,10016992	-3,76654207	0,00016552	0,00198629	**
red vs black	130	mixture	female = male	-1,02165125	0,38873012	-2,62817617	0,0085844	0,10301286	n.s.
red vs black	130	uncolored	female = male	0,85566611	0,06642112	12,8824409	0	0	***
red vs black	1600	red	female = male	-0,40989971	0,14893922	-2,75212736	0,00592095	0,07105139	n.s.
red vs black	1600	black	female = male	-0,16251893	0,10996137	-1,4779638	0,13941747	1	n.s.
red vs black	1600	mixture	female = male	-0,22314355	0,23717075	-0,94085614	0,34677859	1	n.s.
red vs black	1600	uncolored	female = male	0,78035324	0,08254832	9,45329049	0	0	***
